# Datasets2Tools, repository and search engine for bioinformatics datasets, tools and canned analyses

**DOI:** 10.1038/sdata.2018.23

**Published:** 2018-02-27

**Authors:** Denis Torre, Patrycja Krawczuk, Kathleen M. Jagodnik, Alexander Lachmann, Zichen Wang, Lily Wang, Maxim V. Kuleshov, Avi Ma’ayan

**Affiliations:** 1 Department of Pharmacological Sciences, Mount Sinai Center for Bioinformatics, BD2K-LINCS Data Coordination and Integration Center (DCIC), Team Nitrogen of the NIH Data Commons Pilot Project Consortium (DCPPC), Icahn School of Medicine at Mount Sinai, One Gustave L. Levy Place, Box 1603, New York, NY 10029, USA

**Keywords:** Data publication and archiving, Research data, Computational platforms and environments

## Abstract

Biomedical data repositories such as the Gene Expression Omnibus (GEO) enable the search and discovery of relevant biomedical digital data objects. Similarly, resources such as OMICtools, index bioinformatics tools that can extract knowledge from these digital data objects. However, systematic access to pre-generated ‘canned’ analyses applied by bioinformatics tools to biomedical digital data objects is currently not available. Datasets2Tools is a repository indexing 31,473 canned bioinformatics analyses applied to 6,431 datasets. The Datasets2Tools repository also contains the indexing of 4,901 published bioinformatics software tools, and all the analyzed datasets. Datasets2Tools enables users to rapidly find datasets, tools, and canned analyses through an intuitive web interface, a Google Chrome extension, and an API. Furthermore, Datasets2Tools provides a platform for contributing canned analyses, datasets, and tools, as well as evaluating these digital objects according to their compliance with the findable, accessible, interoperable, and reusable (FAIR) principles. By incorporating community engagement, Datasets2Tools promotes sharing of digital resources to stimulate the extraction of knowledge from biomedical research data. Datasets2Tools is freely available from: http://amp.pharm.mssm.edu/datasets2tools.

## Introduction

The introduction of the web enabled several software-based extensions of traditional print publications of research output: a) Research articles can now be published in soft form so they can be more easily copied and distributed; b) Data collected by research studies can be made more easily available to others for reuse and integrative retrospective analyses; and c) Methods and software tools to analyze and visualize research data are becoming more powerful and accessible. While these developments, facilitated by the introduction of the web, have been critical for advancing biomedical research, much work remains to improve how research results are communicated digitally. The introduction of the web also enhanced the field of bioinformatics, in which hundreds of new tools and databases are published yearly. At the same time, the rapid growth in data collection due to the advancement of high-content biotechnologies that extract large amounts of information from biological samples led to the development of centralized biomedical repositories to manage this wealth of data, for example, the Gene Expression Omnibus (GEO)^1^ for transcriptomics and epigenomics data. In fact, there are now many centralized repositories that cover all areas of biomedical and biological research. These repositories are indexed for searching and browsing by sites such as DataMed^2^ and FAIRSharing^3^. Similarly, several efforts have been made to aggregate and index bioinformatics tools, for example, OMICtools^4^.

While there are paths to start from a dataset landing page in a centralized biomedical data repository to generate a publication-ready figure, or an interactive visualization graphic, most centralized biomedical repositories are currently disconnected from the tools that can operate on the datasets contained within them. There are efforts to streamline the process of bioinformatics analysis, but until recently, most bioinformatics tools, once applied to process a dataset, do not provide the user who performed the analysis, with a persistent URL that can subsequently be shared with others. However, web pages that display such pre-generated ‘canned’ analysis results, generated from a dataset contained within a specific centralized data repository, and analyzed by a specific bioinformatics tool, gradually accumulate. Software tools such as GeneMANIA^5^, Enrichr^6,7^, Clustergrammer^8^, and L1000CDS2^9^ provide users who submit gene signatures for analysis, or build interaction networks, with these tools, a persistent URL that can be shared with others. In addition to this, platforms such as Galaxy^10^ provide interfaces that allow users to easily create complex bioinformatics workflows and pipelines that can be collaboratively shared on the web. Similarly, interactive notebooks, such as Jupyter or R Markdown^11^, are platforms by which bioinformatics canned analyses can be systematically shared on the web. These new persistent methods for creating, communicating, and sharing reproducible biomedical and biological research results can be considered a new form of publication we term ‘canned analysis’.

## Results

### Overview

Here we introduce Datasets2Tools, a comprehensive index and platform for the discovery and evaluation of an initial set of 31,473 canned bioinformatics analyses applied to 6,431 datasets via selected tools that provide persistent canned analysis URLs. Datasets2Tools also indexes 4,901 software tools published in one of the four leading bioinformatics journals: *BMC Bioinformatics*, *Bioinformatics*, *Database*, and *Nucleic Acids Research*. Datasets2Tools allows users to rapidly find relevant datasets, tools, and canned analyses digital objects. We anticipate that Datasets2Tools will expand through community contributions. Datasets2Tools is also delivered as a Google Chrome browser extension and an Application Programming Interface (API).

**Figure 1:**
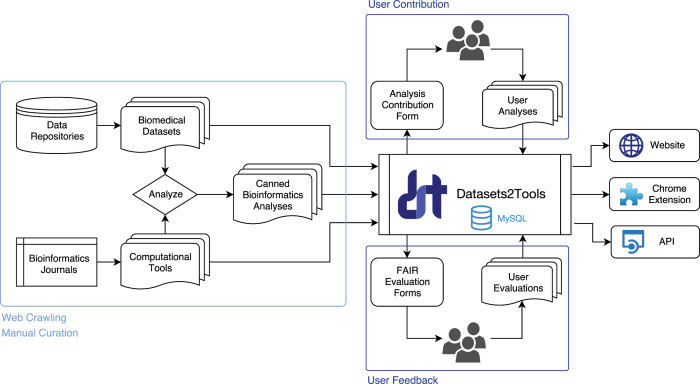
Schematic illustration of the Datasets2Tools components and workflow. Bioinformatics analyses, biomedical datasets, and computational tools are collected through web crawling, API access, and manual curation. These digital objects are indexed within the Datasets2Tools database and made available through a website, a Google Chrome extension, and an API. Users can additionally submit their own analyses and evaluate the FAIRness of indexed digital objects through a form.

One unique feature of Datasets2Tools is the ability to evaluate datasets, tools, and canned analyses by their compliance with the findable, accessible, interoperable, and reusable (FAIR) principles^12^. Users can answer nine Yes or No questions concerning different aspects related to the findability, accessibility, interoperability, and reusability of the three types of digital objects contained within Datasets2Tools: datasets, tools, and canned analyses. Evaluations are stored in a database and displayed as an insignia near each dataset, tool, or canned analysis. Since all indexed bioinformatics tools within Datasets2Tools originate from a publication, the Altmetric Attention Scores and PlumX assessments, as well as PubMed citations for each tool, are listed near the tool’s card. This visualization of tool metrics can assist users to better identify and rank bioinformatics tools. A visual summary of the components and functionality of Datasets2Tools is illustrated in Fig. 1.

### The canned analysis: A new digital object to systematically connect datasets and tools

**Figure 2:**
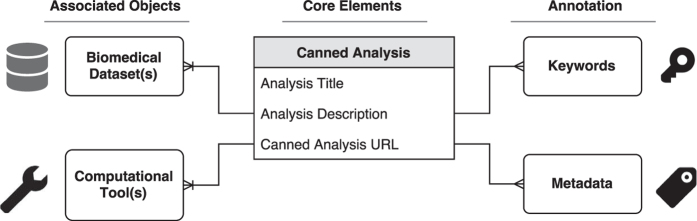
Structure of the canned analysis digital object. The canned analysis is a new type of digital object defined by three sets of components. First, a set of core elements that include a title, a description, and a link to a webpage containing the results of the analysis. Second, references to the biomedical datasets and computational tools used to generate the object. Third, a set of metadata annotations consisting of keywords and structured key-value pairs.

The canned analysis digital object is defined by three key components: 1) a set of core elements, such as a title, a description, and a link to the analysis results; 2) references to the datasets and tools used to generate the analysis; and 3) relevant metadata. A schematic example of the structure of a canned analysis digital object is illustrated in Fig. 2.

**Figure 3:**
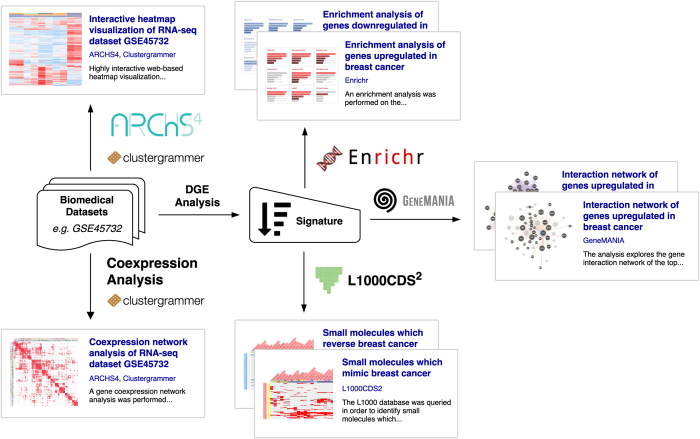
Examples of multiple canned analyses generated from the same dataset. Canned analyses can be used to index information about different types of bioinformatics analyses. The figure shows how a single RNA-seq dataset can be used to generate eight different canned analyses, such as interactive clustered heatmap visualizations and gene expression signature analyses.

The canned analysis URL is the central component of the canned analysis digital object. The URL links to an external webpage that contains the results of the analysis run by the tool(s) on the dataset(s), and that can be viewed by users who navigate to it. References to the datasets and tools used to generate the analyses are made by using the unique accession codes, or names associated with these digital objects. Metadata can be specified as an unstructured set of keywords, or a structured set of key-value term pairs. Such metadata is independent of the datasets and tools used to generate the analysis, allowing customized annotation of individual analyses, as well as interoperability between platforms. Canned analyses can be performed for a wide variety of biomedical and biological data types using a wide variety of bioinformatics tools (Fig. 3).

### The Datasets2Tools database

Many of the canned analyses currently stored in Datasets2Tools were generated from gene expression signatures within the CREEDS resource^13^. These signatures were processed by the Characteristic Direction method^14^ to identify differentially expressed genes, and then up- and down-regulated gene sets were analyzed with Enrichr^6,7^, L1000CDS2^9^, Principal Angle Enrichment Analysis (PAEA)^15^, and GeneMANIA^5^. Another significant portion of the canned analyses currently contained within Datasets2Tools were generated by Clustergrammer^8^ to visualize interactive heatmaps of RNA-seq datasets from GEO processed by ARCHS4^16^. A smaller portion of canned analyses available within Datasets2Tools are datasets generated by the LINCS consortium^17^ and stored within the LINCS Data Portal^18^. For a complete overview of the analyses indexed within the Datasets2Tools database, see Table 1. While these analyses currently cover only one major data repository and a few tools, Datasets2Tools is flexible and scalable, and can support greater variety of canned analyses.

### Exploring digital objects through the Datasets2Tools portal

**Figure 4:**
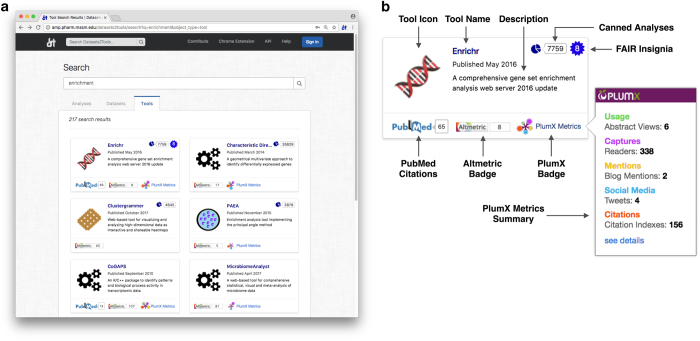
Screenshot of the tool search interface on the Datasets2Tools website. **(a)** Results of a computational tool search for the keyword *enrichment*. Search results are represented as cards, and are sorted by a combination of the tool’s attention metrics, citations, FAIR evaluation results, and number of analyses associated to them. **(b)** Overview of the information described on a tool card for the tool Enrichr. This includes the tool’s name and description, the number of canned analyses generated by the tool, date and citations of the associated publication, Altmetric and PlumX Metrics badges, and a summary of the results of the FAIRness evaluation.

The data stored in the Datasets2Tools database can be browsed through a web interface available at http://amp.pharm.mssm.edu/datasets2tools. The web interface contains a search engine that supports free-text searches, as well as filtering based on keywords and metadata tags. Digital objects returned by search results can be ranked according to several metrics such as date of upload, Altmetric Attention Score of the publication associated with the digital object, and compliance of the digital object with FAIR evaluation (this is further described in the FAIRness evaluations section below). The combination of text search, metadata filtering, and sorting, provides the ability to rapidly identify the digital objects most relevant to the user’s interests. An example of a tool search results webpage is shown in Fig. 4.

After performing a search, users can access information about the list of returned digital objects by navigating to their associated landing pages. Landing pages exist for each dataset, tool, and canned analysis. These landing pages provide summary information about the digital object, including its name, description, accession number, external links, and, where applicable, metadata and links to relevant publications and associated metrics. Landing pages also provide links to the most similar digital objects, as displayed in the ‘Related Objects’ tab. Such similar digital object recommendations are determined by Natural Language Processing (NLP) (further explained in the Methods section).

Landing pages for digital objects of the three types (datasets, tools, and analyses) also display links to other related digital objects that are associated with the specified object through canned analyses. Tool landing pages contain links to the datasets that have been analyzed by the tool, as well as links to all the canned analyses that have been generated by it. Similarly, dataset landing pages contain links of the tools that have been used to analyzed it, as well as links to the corresponding canned analyses. Finally, canned analysis landing pages contain links to the datasets and tools used to generate them.

### Evaluating digital objects based on compliance with FAIRness principles

**Figure 5:**
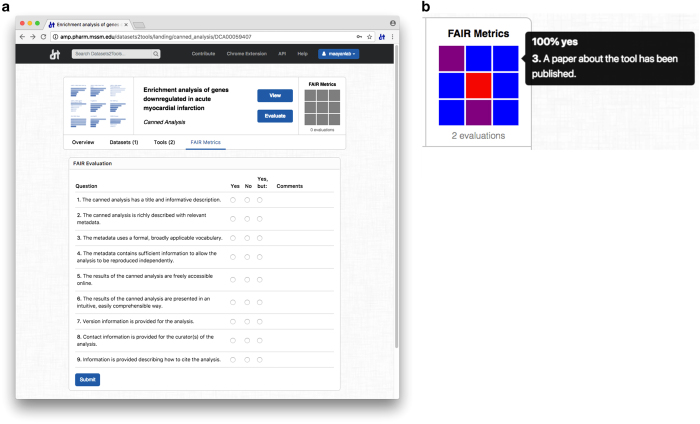
Screenshot of the FAIRness evaluation interfaces for a canned analysis. **(a)** Screenshot of a FAIR evaluation form of a canned analysis, as displayed on an example canned analysis landing page. The form consists of nine Yes or No questions concerning the canned analysis findability, accessibility, interoperability, and reusability. **(b)** Insignia representing the results of the FAIRness evaluations submitted by users for a bioinformatics tool. Each square represents the results of an individual question, and its color ranges from blue (100% positive answers) to red (100% negative answers).

In addition to displaying information stored in the database, landing pages provide easy access to evaluation forms that enable users to grade digital objects according to their compliance with the FAIR principles^12^ (Fig. 5a). Each form consists of a set of nine Yes or No questions, which are designed to address specific aspects of the findability, accessibility, interoperability, and reusability of the digital object type: dataset, tool, or canned analysis. Hence, a different set of questions is provided for each digital object type. A full list of the FAIRness questions is provided in Table 2. These are preliminary suggestive questions that will be reviewed by a governing body that will seek community input. After the user has submitted their evaluation, scores are stored in the database, aggregated with the feedback from all other users, and displayed as a grid insignia on the corresponding landing pages (Fig. 5b). The FAIRness evaluation information is also incorporated within the search ranking system, where users can prioritize and identify resources based on their overall FAIRness score.

### Accessing Datasets2Tools through the API

The Datasets2Tools database can be programmatically accessed through an API. The *search* endpoint provides rapid programmatic access to the data stored in the database. The search API has the same search options as those available from the user search interface, which includes text-based searches, metadata filtering, and sorting based on multiple criteria. The API returns a JSON-formatted string containing information about the digital objects resulting from the search. Detailed documentation of the API is available on the Datasets2Tools website.

### Contributing canned analyses to Datasets2Tools

Datasets2Tools enables users to contribute their own canned analyses to the Datasets2Tools database. This functionality promotes community engagement to make canned analyses more easily findable and accessible. Contributing a canned analysis can be achieved by visiting the Contribute page on the Datasets2Tools website. From there, users can upload their own set of canned analyses through a web-based form, or by submitting a tabular text file, where each line in the file contains information about a single canned analysis based on a template. The information required by the form or template includes the URL of the canned analysis, dataset accession/s, names of associated tools, title and text descriptions, and metadata annotations.

### The Datasets2Tools chrome extension

**Figure 6:**
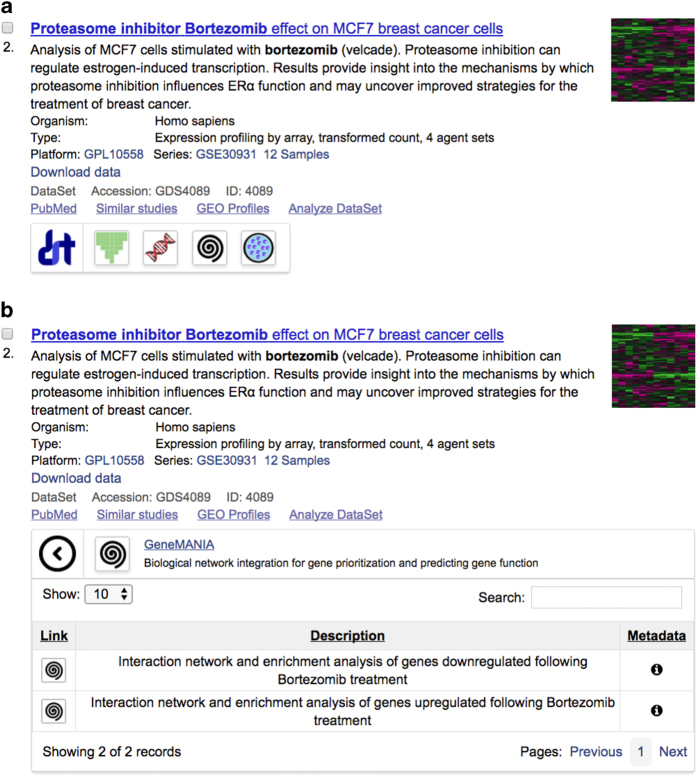
Interface embedded by the Chrome extension on a GEO search results page. **(a)** The Chrome extension embeds toolbars below datasets indexed by Datasets2Tools. The icons on the toolbar represent different tools that have been used to analyze the dataset. **(b)** By clicking on the icons, the user can access the results of the analyses run by the tool applied to the dataset, as well as view metadata associated to the tool, dataset, and canned analysis.

Datasets2Tools is also available as a Chrome extension. The Datasets2Tools Chrome extension can be freely installed onto Google Chrome web browsers from the Google Chrome webstore: https://chrome.google.com/webstore/search/datasets2tools. The Datasets2Tools Chrome extension enhances search results and landing pages of centralized biomedical data repositories such as GEO^1^, DataMed^2^, and the LINCS Data Portal (LDP)^18^ by embedding canned analyses links into search results pages and dataset landing pages. These links provide access to the canned analyses associated to each dataset, displaying information about the canned analyses as either a toolbar for dataset search pages, or as a table for dataset landing pages. A screenshot from the enhanced webpage with the embedded links created by the Datasets2Tools Chrome extension on a GEO search results page is provided in Fig. 6.

## Discussion

Advancements in experimental and computational methods in biomedical research are now producing large volumes of digital data objects that are rapidly accumulating. At the same time, a variety of bioinformatics tools to handle the analysis of all this data are promptly being developed and published. However, systematic linking of digital data objects with the tools that can operate on them is currently lacking; there is a gap between data availability and how much of it is employed for obtaining useful knowledge. *Post hoc* retrospective integrative analyses that utilize already published data are few. At the same time, published analyses from print publications are often difficult to find, access, and reproduce. As far as we know, currently, there is no unified model to allow for the indexing and retrieval of computational canned analyses of biological and biomedical datasets.

Datasets2Tools is a prototype solution for a platform that can enable the systematic management of three types of biomedical digital objects, but it is mostly concerned with systematically indexing a new type of digital object, the canned analysis. The Datasets2Tools model is designed to comply with the FAIR principles to improve the findability, accessibility, interoperability, and reproducibility of tens of thousands of pre-run bioinformatics analyses. Since Datasets2Tools allows users to contribute their own canned analyses to the database, we expect that developers interested in promoting their computational tools will find Datasets2Tools a valuable platform. By uploading analyses generated by the computational methods they develop, bioinformatics developers will potentially reach out to more users unfamiliar with their tools. Furthermore, experimentalists who wish to promote their data analysis pipelines and experimental results may find Datasets2Tools a convenient platform to do so.

The indexing of a large collection of published bioinformatics tools by Datasets2Tools is unique because it integrates information about the tools’ popularity for ranking. For each tool, Datasets2Tools stores the number of citations associated with its publications, as well as Altmetric Attention Scores and PlumX metrics. By displaying such metrics on search result pages, users are able to rapidly identify the most popular and/or similar tools.

Access to the data indexed by Datasets2Tools is provided through a Google Chrome browser extension and an API. The Chrome extension enhances biomedical data repositories by embedding links to canned analyses generated from the datasets indexed within these repositories. In this way, users of the browser extension can extract knowledge more efficiently when interfacing with such repositories. The API allows rapid programmatic access to the data stored within the Datasets2Tools database, allowing users to systematically interface with such data directly from a programming environment.

Finally, Datasets2Tools introduces a method to evaluate digital objects based on their compliance with the FAIR principles. Users of Datasets2Tools can evaluate the FAIRness of datasets, tools, and canned analyses by answering a light-weight questionnaire. Evaluation results are stored in the database and made available on the landing pages of each digital object via a FAIRness insignia. By ranking search results based on FAIRness evaluations, Datasets2Tools allows users to prioritize resources by their compliance with the FAIRness standards. It should be noted that the specifications of the questions in the questionnaire, and the concept of allowing any user to evaluate digital objects stored within the Datasets2Tools database, are *provisional* and are expected to be vetted by a governing body as part of large-scale initiatives such as Big Data to Knowledge (BD2K)^19^, GO-FAIR, and Elixir (https://www.elixir-europe.org). Moreover, FAIRness assessments can be automated by simply examining the compliance of a digital object within the Datasets2Tools database with the FAIR criteria. The effort placed forth to design and implement Datasets2Tools is contributing toward realizing the vision of the NIH Data Commons^20^.

## Methods

### Database schema

Information about biomedical datasets, computational tools, and canned analyses is indexed in a relational MySQL database. For each dataset, the following information is stored: repository of origin, accession, title, description, and landing page URL. For each tool, the following information is stored: tool name, description, homepage URL, associated publication(s), Altmetric Attention Score, and number of citations for each publication. Canned analyses are defined as a many-to-many relationship between biomedical datasets and computational tools. For each canned analysis, the following information is stored: dataset(s) and tool(s) used for its generation, title, description, results URL, and an arbitrary number of unstructured (keywords) or structured (key-value pairs) metadata elements.

### Annotation of datasets

To supplement information about datasets indexed within Datasets2Tools, NCBI eSearch and eSummary API, and the LINCS Data Portal API, were used to query these resources to obtain metadata. Dataset metadata is then uploaded to the Datasets2Tools database and displayed on the Datasets2Tools website.

### Natural language processing to compute similarity among datasets, tools, and analyses

Recommendations for similar bioinformatics software tools, canned analyses and analyzed datasets digital objects were generated by a Natural Language Processing (NLP) approach with the nltk and sklearn Python libraries. First, the text descriptions of each abstract describing a software tool or a dataset were extracted, tokenized, and processed by removing the most common stopwords in the English language. Next, Term Frequency-Inverse Document Frequency (TF-IDF), a leading method for text-based recommender systems^21^, was applied on the processed corpus. This resulted in the generation of an M x N weight matrix representing the relative importance of M words for each of N datasets, software tools, or canned analysis descriptions. Finally, cosine similarity was applied on the weighted matrix to calculate the pairwise similarity between descriptions. The top 10 datasets, tools, or canned analyses with the most similar descriptions are reported at the bottom of each corresponding landing page on the Datasets2Tools website.

### Collecting and indexing bioinformatics tools

Information about bioinformatics tools was obtained by downloading the titles and abstracts of articles published since 2010 in four leading journals in the field of bioinformatics: *BMC Bioinformatics*, *Bioinformatics*, *Database*, and *Nucleic Acids Research*. Tool names, tool descriptions, and links to the tool’s homepage were obtained by mining the text from publication titles and abstracts. Information about the article’s citations was obtained by searching the article DOI using the NCBI PubMed eSearch API, while information about the tool’s Attention score was obtained by searching the article describing the tool’s DOI using the Altmetric API. Information about newly published computational tools and the metrics of all indexed tools is updated on a weekly basis.

### Development of FAIRness evaluation forms

The FAIRness evaluation platform is comprised of three different forms, each consisting of a set of nine Yes or No questions. Each form is specifically designed to address the FAIRness of the dataset, tool, or canned analysis in question. The results of user FAIRness evaluations are stored in the MySQL database and displayed as colored grids on the dataset landing pages. Grids are generated using the JavaScript library D3.JS^22^.

### Development of the Datasets2Tools website and Google Chrome extension

The back-end of the website was developed with Flask^23^; the front-end of the website was developed with JavaScript, HTML, and CSS. The Chrome extension was developed using JavaScript, HTML, and CSS. Upon loading a search, or a dataset landing page, on one of the supported data repositories, the Datasets2Tools Chrome extension scrapes the webpage in order to extract the accessions numbers of the datasets displayed. The script subsequently queries the Datasets2Tools database in order to obtain information about the canned analyses associated with each dataset. The extension then displays the information about available canned analyses on the repository’s webpage through an intuitive interface.

## Additional information

**How to cite this article:** Torre, D. *et al.* Datasets2Tools, repository and search engine for bioinformatics datasets, tools and canned analyses. *Sci. Data*. 5:180023 doi: 10.1038/sdata.2018.23 (2018).

**Publisher’s note:** Springer Nature remains neutral with regard to jurisdictional claims in published maps and institutional affiliations.

## Figures and Tables

**Table 1:** Summary of the canned analyses currently indexed by Datasets2Tools.

**Source Repository**	**Dataset Type**	**Tool Name**
Gene Expression Omnibus (6,431 datasets)	Microarray (1,934 datasets)	Enrichr (7,758 analyses)
		L1000CDS2 (7,756 analyses)
		PAEA (3,879 analyses)
		GeneMANIA (7,435 analyses)
	RNA-seq (4,497 datasets)	ARCHS4 (4,645 analyses)
LINCS (94 datasets)	KINOMEscan (65 datasets)	Interactive data browsers (11 analyses)
	Fluorescence imaging cell count assay (12 datasets)	
	Fluorescence imaging apoptosis assay (4 datasets)	
	RPPA protein state assay (4 datasets)	
	ELISA protein state assay (2 datasets)	

**Table 2:** Summary of the FAIR evaluation questions.

***#***	**Question**	**Object Type**	**FAIR**
1	The canned analysis has a title and informative description.	Analysis	F,R
2	The canned analysis is richly described with relevant metadata.	Analysis	F,I,R
3	The metadata uses a formal, broadly applicable vocabulary.	Analysis	F,I,R
4	The metadata contains sufficient information to allow the analysis to be reproduced independently.	Analysis	R
5	The results of the canned analysis are freely accessible online.	Analysis	A
6	The results of the canned analysis are presented in an intuitive, easily comprehensible way.	Analysis	A,R
7	Version information is provided for the analysis.	Analysis	I,R
8	Contact information is provided for the curator(s) of the analysis.	Analysis	R
9	Information is provided describing how to cite the analysis.	Analysis	R
1	A standardized ID or accession number is used to identify the dataset.	Dataset	F,R
2	The dataset is described with metadata using a formal, broadly applicable vocabulary.	Dataset	F,I,R
3	Information is provided on the experimental methods used to generate the data.	Dataset	R
4	The dataset is hosted in an established data repository, if a relevant repository exists.	Dataset	F,A
5	The dataset can be downloaded for free from the repository.	Dataset	A
6	Version information is provided for the dataset.	Dataset	R
7	Contact information is provided for the creator(s) of the dataset.	Dataset	R
8	Information is provided describing how to cite the dataset.	Dataset	R
9	Licensing information is provided on the dataset’s landing page.	Dataset	R
1	The tool has a unique name and an informative description.	Tool	F,R
2	The tool can be freely downloaded or accessed from a webpage.	Tool	F,A
3	A paper about the tool has been published.	Tool	R
4	Tutorials for the tool are available on the tool’s homepage.	Tool	R
5	Source code is shared in a public repository and is documented.	Tool	R
6	Previous versions of the tool are made available.	Tool	F,I,R
7	Contact information is provided for the creator(s) of the tool.	Tool	R
8	Information is provided describing how to cite the tool.	Tool	R
9	Licensing information is provided on the tool’s homepage.	Tool	R
Three sets of FAIR evaluation questions are designed for each of the three types of digital objects indexed by Datasets2Tools. These questions are related to the FAIRness evaluation of each type of digital object: tool, dataset, or canned analysis.			
